# Development and Testing of an Integrated Rotating Dynamometer Based on Fiber Bragg Grating for Four-Component Cutting Force Measurement

**DOI:** 10.3390/s18041254

**Published:** 2018-04-18

**Authors:** Mingyao Liu, Junjun Bing, Li Xiao, Kang Yun, Liang Wan

**Affiliations:** 1 School of Mechanical and Electronic Engineering, Wuhan University of Technology, Wuhan 430070, Hubei, China; lmylyf@126.com (M.L.); whutbingo@163.com (J.B.); 15071221564@163.com (L.W.); 2 Jianghan Machinery Research Institute Co., Ltd., CNPC, Jingzhou 430024, Hubei, China; XiaoL@cnpc.com.cn

**Keywords:** cutting force, rotating dynamometer, fiber Bragg grating (FBG), arcuate beam

## Abstract

Cutting force measurement is of great importance in machining processes. Hence, various methods of measuring the cutting force have been proposed by many researchers. In this work, a novel integrated rotating dynamometer based on fiber Bragg grating (FBG) was designed, constructed, and tested to measure four-component cutting force. The dynamometer consists of FBGs that are pasted on the newly designed elastic structure which is then mounted on the rotating spindle. The elastic structure is designed as two mutual-perpendicular semi-octagonal rings. The signals of the FBGs are transmitted to FBG interrogator via fiber optic rotary joints and optical fiber, and the wavelength values are displayed on a computer. In order to determine the static and dynamic characteristics, many tests have been done. The results show that it is suitable for measuring cutting force.

## 1. Introduction

Nowadays, real-time on-line monitoring of cutting force is an essential requirement in the machining processes. As one of the most significant machining process variables, cutting force can be used to optimize cutting parameters, improve cutting conditions, investigate cutting tools performance, predict the surface roughness, monitor tool wear or failure, and others.

Commonly, studies about cutting force measurement mainly focus on direct measurement and indirect measurement of cutting force. Indirect measurement always detects the power or current of the spindle motor or the driving motor to reflect the cutting force [1,2,3,4]. Besides, some researchers work on measuring the displacement of the machining tool spindle [5,6]. The indirect measurement has the advantages of simple structures and easy installation. However, the signals from electrical transducers are susceptible to forces of temperature, transmission, mechanical structure, and so on, so it is difficult to measure the cutting force precisely. Direct measurement of cutting force can be divided into two parts according to the installation, which are clamped on the table and mounted on the spindle. No matter what the dynamometer is, it usually utilizes an elastic structure to convert cutting force into strain, and then detects the strain by sensing elements. In table dynamometers, elastic structures include the octagonal ring [7,8], the oval octagonal ring [9], the ring [10], and so on. For rotating dynamometers mounted on the spindle, elastic structures consist of a cylindrical deformation beam [11], Γ beam-type [12,13], E-type diaphragm [14,15] and others. In contrast to table dynamometers, rotating dynamometers have several advantages, such as less intermediate force transmission components, allowing for various cutting tools sizes and force configurations. From the sensing elements, there are capacitive [11], strain gauge [13,14,15,16], piezoelectric [17,18,19], fiber Bragg grating (FBG) [10,20,21], and so on. Capacitors and strain gauges are susceptible to electromagnetic interference and their processing circuits are complex. Piezoelectric materials have high demand for ambient temperature and humidity. However, the cutting force test environment is filled with humidity and magnetic fields, in which capacitors, strain gauges, and piezoelectric materials are not suitable. FBG is not sensitive to humidity and magnetic fields, and is resistant to corrosion, small in volume, and can measure multiple points in one optical fiber. Therefore, FBG is more applicable for cutting force measurement. Reference [12,22] designed a spindle-integrated method for cutting force measurement based on FBG, realizing the measurement of three-axis forces, but not measuring the torque.

In this paper, a novel integrated rotating dynamometer based on fiber Bragg grating for four-component cutting force measurement has been proposed. Firstly, the elastic structure used in this system is introduced as two mutual-perpendicular semi-octagonal rings which are convenient for pasting FBGs. Secondly, the layout of the FBGs is specially selected under the analysis of finite element method (FEM) simulation results and can realize the decoupling of four-component cutting force in theory. Static calibration and dynamic experiments were undertaken to evaluate the performance of the dynamometer. Test results indicate that the developed dynamometer is qualified to measure the four-axis loads in milling or drilling processes. 

## 2. Experimental Section

### 2.1. Design Principle

**Figure 1:**
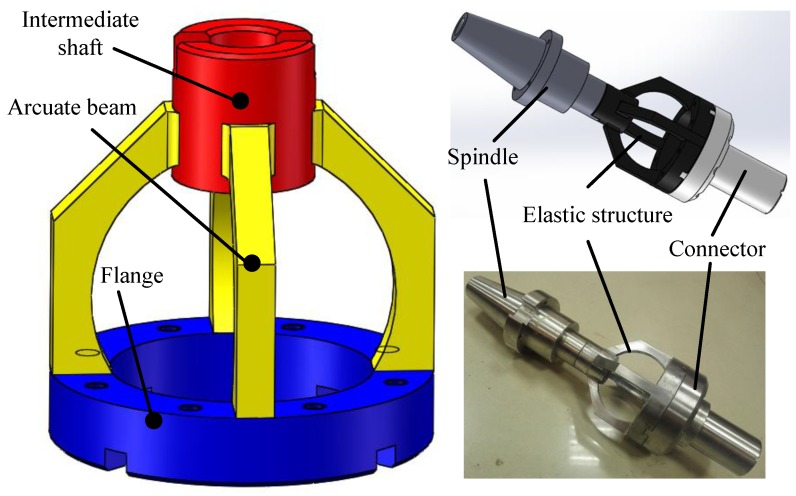
The measurement structure.

The purpose of this study aimed at developing an integrated rotating dynamometer to measure four-axis loads (*viz*. $F_{x},F_{y},F_{z},T$) for milling or drilling processes. A novel elastic structure of two mutual-perpendicular semi-octagonal rings has been put forward, as depicted in Figure 1. The measurement structure consists of the spindle, elastic structure, and connector. The spindle connects with the machine head and the connector joins the tools. But, this connector is designed to be applied to the multi-dimensional force device. The elastic structure is made up of the intermediate shaft, the arcuate beam, and the flange. The arcuate beam is used to convert cutting force into strain. When the four-axis loads act on the end of the connector, the four arcuate beams produce deformation and stress.

**Figure 2:**
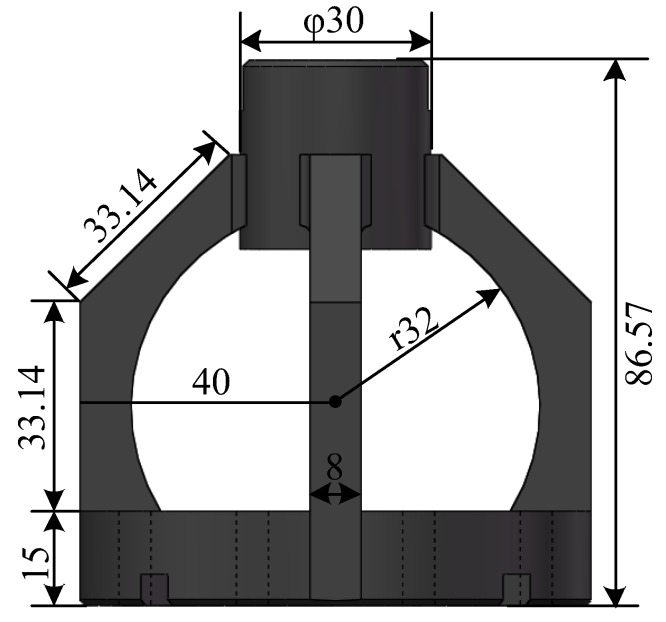
The size of the elastic structure.

In order to obtain a larger range of the measurement, we choose 40CrNiMoA steel as the material of the elastic structure; its elastic modulus, poisson ratio, and yield strength are 210 GPa, 0.3, and 835 MPa, respectively. The material of the spindle and the connector is stainless steel 304. The size of the elastic structure is shown in Figure 2.

### 2.2. Sensor Design

When a broad-band light enters the fiber Bragg grating through the optical fiber, the narrow-band light of a particular wavelength is reflected back. The reflected light satisfies the Bragg scattering conditions. It is expressed by the following equation.

(1) $$\lambda_{B}=2n_{eff}\Lambda$$

where *λ_B_* is the Bragg wavelength, *n_eff_* is the effective refractive index of the fiber core, and Λ is the grating period. The wavelength of a FBG is influenced by strain and temperature simultaneously. Since the experiment of the study is carried out at room temperature, this paper doesn’t consider the impact on the dynamometer. The equation can be given by

(2) $$\frac{\Delta \lambda_{B}}{\lambda_{B}}=(1-p_{e})\varepsilon$$

where *P_e_* is the effective photo-elastic coefficient; generally, the value of *P_e_* is 0.22; Δ*λ_B_* is the change of wavelength; *ε* is the strain.

**Figure 3:**
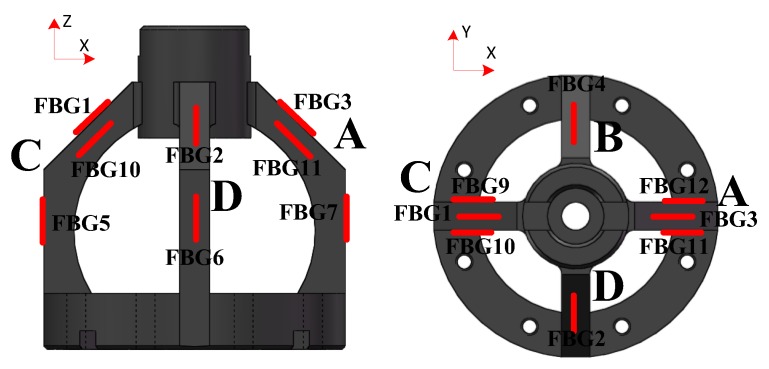
The layout of fiber Bragg gratings (FBGs).

As it is difficult to get the theoretical formula to calculate the surface stress of the elastic structure, the finite element method (FEM) was used to investigate the distribution of the elastic structure and select the layout of FBGs. In order to achieve a larger sensitivity and decrease the cross-interference influence, the location of the FBGs on the force sensing element is shown in Figure 3. FBGs 1–12 are pasted on the surfaces of the elastic structure by modified acrylic adhesive. Different FBGs can be connected with one optical fiber. When four-component cutting force acts on the dynamometer, the surfaces of the elastic structure produce strains. Besides, the wavelengths of FBGs change along with the surface strains of the elastic structure. And then, the information of wavelengths is transmitted to the FBG interrogator through optical fiber. The wavelengths of FBGs are demodulated by the interrogator and are displayed on the computer. At last, the relationship between the wavelength changes of the FBGs and the four-component cutting forces is established. The X-direction force $F_{x}$ is detected by FBG1 and FBG3; the Y-direction force $F_{y}$ is supported by FBG2 and FBG4; the Z-direction force $F_{z}$ is described by FBG5-8; the torque T is represented by FBG9-12. The self-decoupling relation is shown in Equation (3).

When the force $F_{x}$ acts on the tool holder, the wavelengths changes of FBG1 and FBG3, FBG5 and FBG7 are equal in magnitude and opposite in direction; the wavelengths changes of FBG9 and FBG10, FBG11 and FBG12 are equal in magnitude and direction. The wavelengths of FBG2, FBG4, FBG6, and FBG8 are almost unchanged because of their locating in the neutral layer. So, under the force $F_{x}$, the strains of FBG2 and FBG4, the strains of FBG5–8, and the strains of FBG9–12 cancel each other out through Equation (3). When the force $F_{y}$ is applied to the tool holder, the effect is similar to force $F_{x}$. When the force $F_{z}$ is loaded, the wavelength changes of FBG1–FBG4 are equal in magnitude and direction to FBG5–8 and FBG9–12. So, under the force $F_{z}$, the strains of FBG1 and FBG3, the strains of FBG2 and FBG4, and the strains of FBG9–12 cancel each other out through Equation (3). When the torque T acts on the tool holder, the wavelengths of FBG1–4 and FBG5–8 are almost unchanged because of their location in the neutral layer. The wavelengths changes of FBG9 and FBG10 are equal in magnitude and direction to FBG11 and FBG12. So, under the torque T, the strains of FBG1 and FBG3, the strains of FBG2 and FBG4, and the strains of FBG5-8 cancel each other out through Equation (3). Therefore, every force and torque at any time can be measured by Equation (3) due to the structure’s symmetry.

(3) $$\left\{ \begin{array}{l} F_{x}=k_{1}\frac{\varepsilon_{1}-\varepsilon_{3}}{2}=k_{1}\varepsilon_{F_{x}}\\ F_{y}=k_{2}\frac{\varepsilon_{2}-\varepsilon_{4}}{2}=k_{2}\varepsilon_{F_{y}}\\ F_{z}=k_{3}(\varepsilon_{5}+\varepsilon_{6}+\varepsilon_{7}+\varepsilon_{8})=k_{3}\varepsilon_{F_{z}}\\ T=k_{4}\frac{\varepsilon_{9}-\varepsilon_{10}+\varepsilon_{11}-\varepsilon_{12}}{4}=k_{4}\varepsilon_{T} \end{array}\right.$$

Although the experiment of the study is conducted at room temperature, temperature compensation must be discussed considering the actual processing environment. Since the heat conducts from the tool, it has the same effect on FBG1–4, as on FBG5–8 and FBG9–12. Therefore, temperature effects can be offset by subtraction in the X-direction, Y-direction, and T-direction through Equation (3). As for the Z-direction, a free FBG is located near FBG5–8 as a reference for eliminating the interference of temperature. Besides, TCFBGs can be used for temperature compensation [23].

### 2.3. Theoretical Analysis of Static Properties

**Figure 4:**
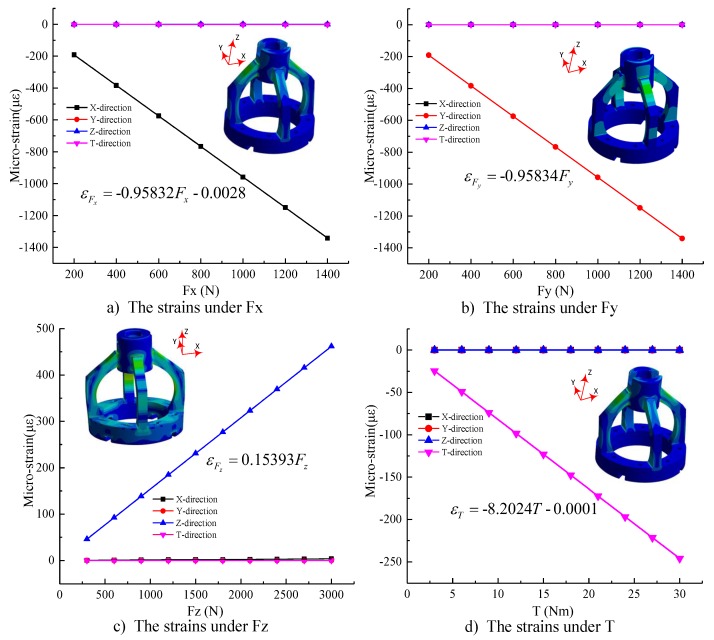
The strains under the four-axis loads.

ANSYS Workbench (ANSYS Inc., Pittsburgh, PA, America) was used to determine the strain distributions of the arcuate beams subjected to each load. The measurement structure used in this simulation is “Hex Dominant”; the volume mesh size of the elastic structure is 1 mm and the volume mesh sizes of the spindle and the connector are 2 mm. According to analysis of the structural strength of the dynamometer by ANSYS Workbench, the dynamometer is designed to measure cutting force up to 1400 N in X-direction and Y-direction, 3000 N in Z-direction, and torque up to 30 Nm. When the dynamometer is under the maximum of $F_{x}$, $F_{y}$, $F_{z}$, and ${}_{T}$ simultaneously, the result shows the maximum equivalent (Von-Mises) stress is 573.15 MPa, which is less than the yield strength of the 40CrNiMoA material of 835 MPa. Considering the real constraint and load conditions, the spindle was selected to fix the dynamometer to and the connector was designed to endure the X-direction force $F_{x}$, the Y-direction force $F_{y}$, the Z-direction force $F_{z}$, and the torque T individually. The static calibration loads of simulation are based on full scale output (FSO) with an incremental step of 200 N in X, Y-direction force, 300 N in Z-direction force, and 3 Nm torque. The strains of FBGs through the simulation are recombined by Equation (3), and the calibration curves of four-axis loads were achieved, as shown in Figure 4a–d. 

It is known from the fitting line of Figure 4 that the force sensing element can be considered as a linear system. So, the relationship can be described as Equation (4) (Figure 4).

(4) $$\left[\begin{array}{l} \varepsilon_{F_{x}}\\ \varepsilon_{F_{y}}\\ \varepsilon_{F_{z}}\\ \varepsilon_{T} \end{array}\right]{=10}^{-3}\cdot \left[\begin{array}{cccc} -958.32 & 0.01 & 1.16 & -0.51\\ 0.02 & -958.34 & 0 & -0.15\\ -0.04 & -0.05 & 153.93 & 0.18\\ -1.41 & -0.04 & 0 & -8202.4 \end{array}\right]\left[\begin{array}{c} F_{x}\\ F_{y}\\ F_{z}\\ T \end{array}\right]+\left[\begin{array}{c} -0.00245\\ -0.00015\\ -0.00023\\ -0.01145 \end{array}\right]$$

### 2.4. Experimental Device Introduction

**Figure 5:**
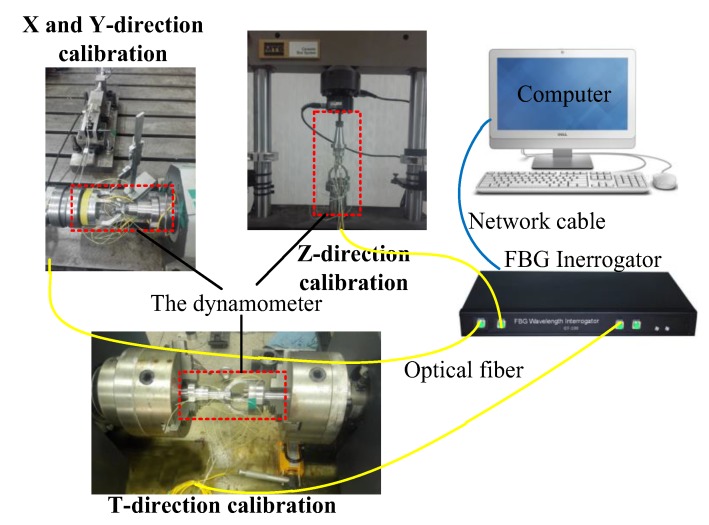
Static calibration device.

A static calibration test was performed to determine the static properties of the dynamometer in four directions, namely $F_{x}$, $F_{y}$, $F_{z}$, and T, respectively, as shown in Figure 5. The calibration tests of the X-direction and Y-direction are operated on the spindle rotation experimental platform which was designed by ourselves. Before the X-direction calibration test, the arcuate beam A was adjusted to be horizontal using a Vernier caliper. After that, the force $F_{x}$ is loaded by the hand wheel of the radial force device. When calibrating the Y-direction, we rotated the dynamometer 90 degrees and used the same calibration method as for the X-direction. The Z-direction calibration test was carried out on an MTS (Eden Prairie, MN, USA) static universal material testing machine. The T-direction calibration test is done by the microcomputer-controlled material torsion test machine. The output wavelengths of FBGs under cutting forces are all recorded by the FBG interrogator.

**Figure 6:**
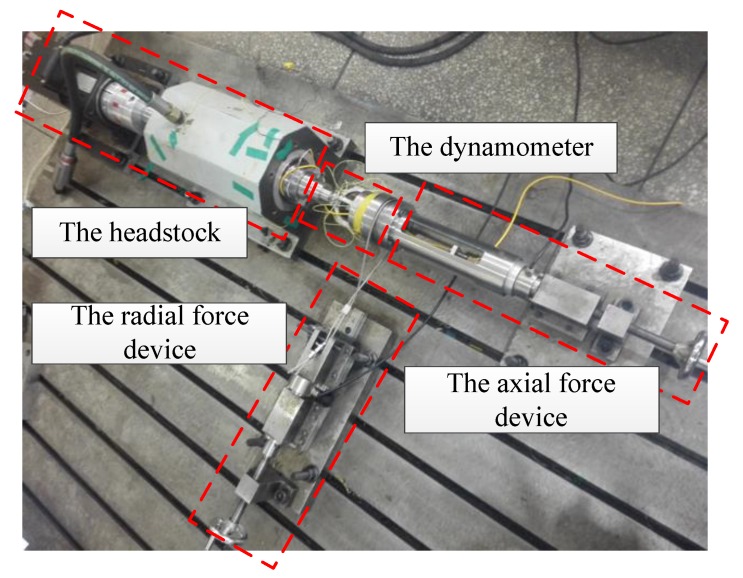
The dynamic experimental device.

Considering the fact that cutting force is not static during the cutting process, the impacting modal test and the dynamic test should be performed to identify the dynamic performance of the dynamometer. The dynamometer was excited by using a modal impact hammer, and an accelerometer was connected to the arcuate beam of the dynamometer in the impacting modal test. The dynamic test was completed by simulating loading cutting forces on the spindle rotation experimental platform as depicted in Figure 6. The experimental platform consists of the headstock and the force device. The headstock is made up of the servo motor and the spindle housing. The force device is composed of the radial force device and the axial force device. The force device is used to simulate the actual loading of cutting force. The cutting forces $F_{x}$, $F_{y}$ are loaded by the radial force device and the cutting force $F_{z}$ is loaded by the axial force device.

## 3. Results and Discussion

### 3.1. Static Calibration

**Figure 7:**
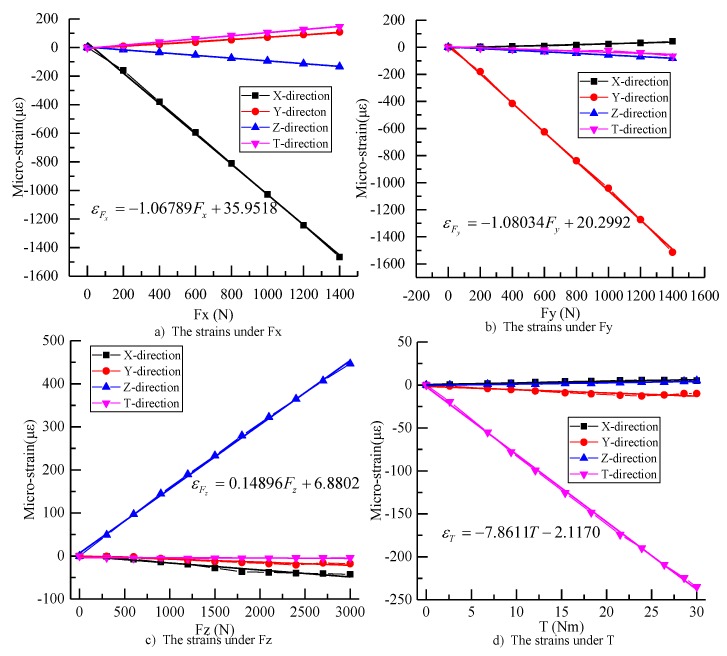
The strains under the four-axis loads.

The calibration test is a process to determine the relationship between the input and output data. The output wavelengths of the dynamometer were recombined and calculated by using Equations (2) and (3), and the calibration curves of the X-direction force $F_{x}$, the Y-direction force $F_{y}$, the Z-direction force $F_{z}$, and the torque ${}_{T}$ were achieved, as shown in Figure 7a–d.

According to the calibration lines, it is obvious that the dynamometer’s sensitivities were about −1.06789 με/N, −1.08034 με/N, 0.14896 με/N, and −7.8611 με/Nm. The differences of sensitivities are 11.43%, 12.73%, −3.23%, and −4.16%, in contrast to the sensitivities of simulation analysis. The sensitivity differences of the X-direction and the Y-direction are relatively large.

Besides, the coefficient of determination for the calibration lines is basically above 0.9. The matrix of the static calibration can be achieved by the least square method. Equation (5) shows the relationship between loads and strains.

(5) $$\left[\begin{array}{c} \varepsilon_{F_{x}}\\ \varepsilon_{F_{y}}\\ \varepsilon_{F_{z}}\\ \varepsilon_{T} \end{array}\right]{=10}^{-3}\cdot \left[\begin{array}{cccc} -1067.89 & 30.55 & -16.18 & 188.35\\ 81.68 & -1080.34 & -7.12 & 376.56\\ -96.08 & -59.47 & 148.90 & 154.73\\ 107.51 & -40.27 & -0.57 & -7861.1 \end{array}\right]\left[\begin{array}{c} F_{x}\\ F_{y}\\ F_{z}\\ T \end{array}\right]+\left[\begin{array}{c} 31.86\\ 10.65\\ 11.80\\ -6.19 \end{array}\right]$$

Cross-interference, defined as the ratio of sensor output in lateral directions to the one when force in primary direction, is an important factor for precise applications requiring high accuracy. To assess the cross-interference in four directions, some tests were done. Some maximum loads were applied to the dynamometer in the four directions, and the outputs of the FBGs were obtained and then substituted to Equation (5) to calculate the forces. By comparing the force values between applied and calculated, the decoupling errors can be calculated, as shown in Table 1. The results showed the measurement errors were small with a maximum of −2.10%. The maximum cross-interference error was −5.35%, which occurred for the Y-direction force $F_{y}$ to torque ${}_{T}$, and all the others did not exceed 4%. It means that the dynamometer is acceptable for use in cutting force measurement.

### 3.2. Natural Frequency Identification

**Figure 8:**
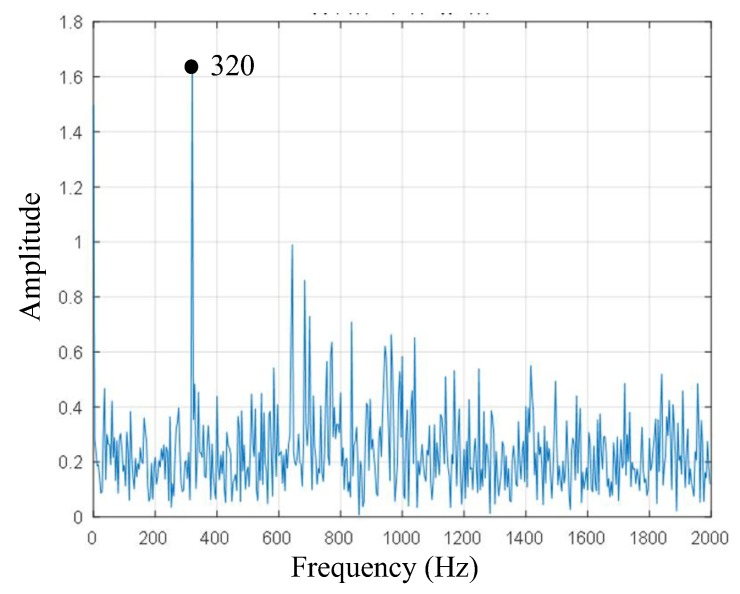
First-order frequency of impacting modal test.

In order to ensure the stability of the machining process, the natural frequency of a sensor should be four times larger than the frequency of the machine tool’s exciting vibration [24]. The first-order natural frequency was 320 Hz, as shown in Figure 8. Hence, the dynamometer can fulfill real-time cutting force measurement when the spindle speed is less than 320 × 0.25 × 60 = 4800 r/min.

### 3.3. Dynamic Characteristic Analysis

In order to simulate the actual processing conditions, dynamic experiments were carried out. Since the spindle was in the process of rotation, 12 FBGs were divided into three groups, namely FBG1–4, FBG5–8, and FBG9–12. In this experiment, the spindle speeds were 100 r/min, 200 r/min, and 300 r/min, respectively. The applied forces in the radial direction (X and Y directions) were 400 N, 800 N, and 1200 N, respectively, and the applied forces in Z direction were 500 N, 1000 N, and 1500 N, respectively. The obtained wavelengths were fitted with sinusoidal functions over time. The quality of the fitted curves was described by the “coefficient of determination”. The closer the coefficient is to 1, the stronger the explanatory power of the equation’s variable to y is, and the better the fitting of the model to the data is. Moreover, the characteristics of the fitting curves were represented by the amplitude, angular velocity, and intercept.

**Figure 9:**
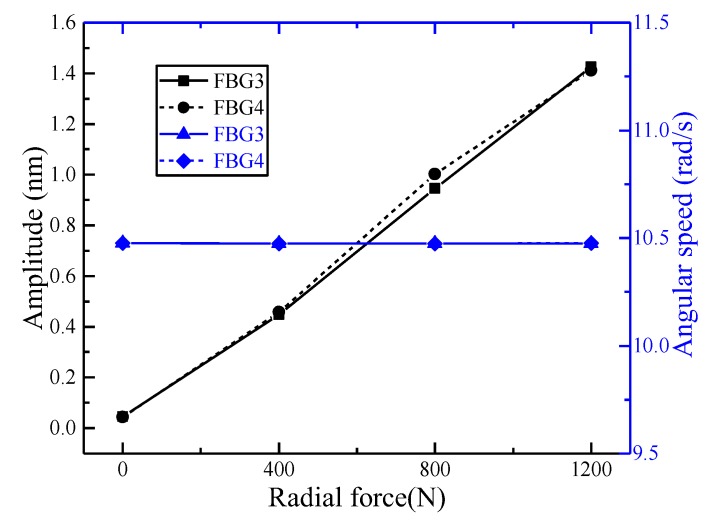
The characteristics of FBG3 and 4 at 100 r/min.

**Figure 10:**
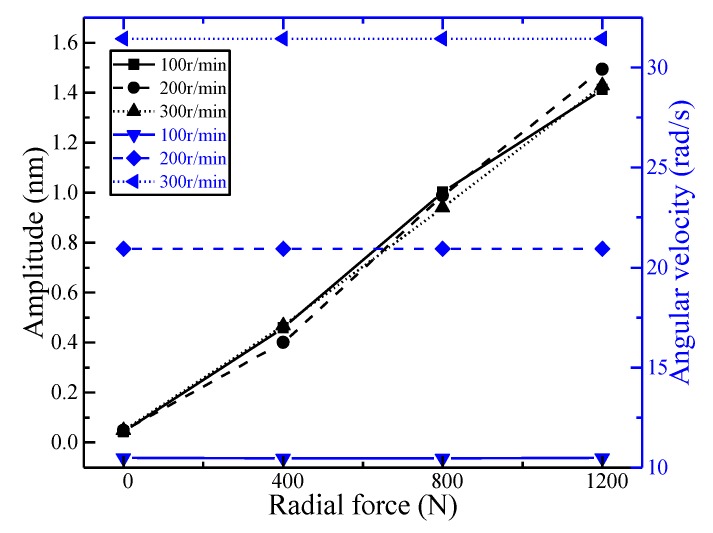
The characteristics of FBG4 at different spindle speeds.

When carrying out radial loading tests, the first group (FBG1–4) was analyzed. When the spindle speed was 100 r/min and the loading forces were 400 N, 800 N, and 1200 N, respectively, taking FBG3 and FBG4 as an example, the amplitude and angular velocity were expressed as illustrated by Figure 9. As the fitting coefficients were above 0.98, we could see that the fitting effect was good. The amplitude and angular velocity of FBG3 and FBG4 have little difference under different forces. Therefore, FBG3 and FBG4 can be regarded as the same class. In the same way, FBG4 can be used as an example to analyze the relationship between the amplitude and angular velocity of FBG1–4 at different speeds and different forces. As illustrated in Figure 10, the amplitude of fitting curves at 100 r/min, 200 r/min, and 300r/min were basically the same under the same radial force, and the angular velocities basically meet the 1 times, 2 times, and 3 times relationship; the angular velocities of the fitting curves were basically the same under the same rotational speed and different radial forces, and the amplitudes basically satisfy the relations of 1 times, 2 times, and 3 times. The features of the second group (FBG5–8) and the third group (FBG9–12) were similar to those of the first group (FBG1–4) at different speeds and different sizes of radial forces.

**Figure 11:**
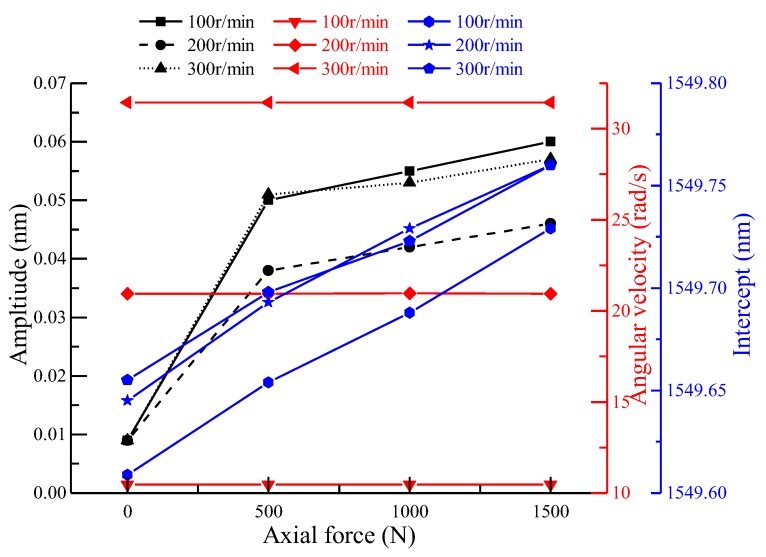
The characteristics of FBG4 under different spindle speeds.

When carrying out the axial loading test, the second group (FBG5–8) was analyzed. When the spindle speed was 100 r/min and the loading forces were 500 N, 1000 N, and 1500 N, respectively, taking FBG6 as an example to analyze the relationship between the amplitude, angular velocity, and intercept of FBG5–8 at different speeds and different forces, the results were as shown in Figure 11. As the fitting coefficients were above 0.99, except when there was no external axial force, the fitting effect was good. The intercepts of the fitting curves at 100 r/min, 200 r/min, or 300 r/min basically showed an increasing tendency, which was consistent with the increasing of the forces. The angular velocities of the fitting curves were basically the same under the same rotational speed. The amplitude of the fitting curves basically showed an increasing trend. The reason was that the loading axis didn’t coincide with the axis of the main shaft. The features of the first group (FB1–4) and the third group (FBG9–12) were similar to those of the second group (FBG5–8) at different speeds and different forces.

## 4. Conclusions

A novel integrated rotating dynamometer based on fiber Bragg grating for four-component cutting force measurement has been developed and tested. The corresponding theoretical analysis has been completed to support structural design and the layout of FBGs. Static and dynamic tests were done in order to evaluate the performance of the developed rotating dynamometer. The results showed that sensitivities were 1.06789 με/N, 1.08034 με/N, 0.14896 με/N, and 7.8611 με/Nm, respectively, with low cross-sensitivity errors below 5.35%. The results of dynamic analysis showed that the first-order natural frequency was approximately 320 Hz. The simulation processing experiment indicated that the dynamometer could operate at the actual processing conditions.

## Figures and Tables

**Table 1:** The results of cross-interference.

Axes	Load	Output after Decoupling				Error (%)			
		Fx (N)	Fy (N)	Fz (N)	T (Nm)	Fx	Fy	Fz	T
Fx (N)	1400	1403.7	33.05	−9.31	−0.88	0.26	2.38	−0.31	−3.00
Fy (N)	1400	50.87	1389.0	−62.18	−1.57	3.62	−0.79	−2.09	−5.35
Fz (N)	3000	26.82	9.43	2975.0	−0.21	1.91	0.68	−0.83	−0.72
T (Nm)	30	31.93	31.98	−43.06	29.37	2.27	2.30	−1.45	−2.10
